# People powered research: what do communities identify as important for happy and healthy children and young people? A multi-disciplinary community research priority setting exercise in the City of Bradford, United Kingdom (UK)

**DOI:** 10.1186/s12939-023-01881-y

**Published:** 2023-04-25

**Authors:** Christopher Cartwright, Aamnah Rahman, Shahid Islam, Bridget Lockyer, Euroline Roper, Meegan Worcester, Melany Zarate, Rosemary McEachan, on behalf of the Happy and Healthy Children Steering Group, Nadera Amini, Ruby Hammard, Peter Horner, Halima Iqbal

**Affiliations:** Born in Bradford, Braford Institute for Health Research, Bradford Teaching Hospitals NHS Foundation Trust, Duckworth Lane, Bradford, BD9 6RJ UK

**Keywords:** Priority setting, Born in Bradford, Health, Children, Ethnicity, Patient and public involvement, Co-production

## Abstract

**Background:**

Involving communities in research priority setting can increase the relevance and efficiency of research, leading to better health outcomes. However these exercises often lack clarity in how communities are involved and the extent to which priorities are acted upon is unclear. Seldom-heard groups, for example ethnic minorities may experience barriers to participation. We report methods and outcomes of an inclusive co-produced community research priority setting exercise within the multicultural and deprived city of Bradford, UK. The aim was to identify priorities for keeping children happy and healthy and was undertaken by the Born in Bradford (BiB) research programme to inform future research agendas.

**Methods:**

A 12 member multi-disciplinary, multi-ethnic community steering group led the process using a modified James Lind Alliance approach between December 2018-March 2020. Research priorities were collected through a widely distributed paper and online survey. Respondents were asked to list three important things to keep children i) happy, ii) healthy and what needs to change to improve either health or happiness. Free text data were coded iteratively by community researchers, and shared priorities were co-produced in a series of workshops and meetings with the community steering group and community members.

**Results:**

Five hundred eighty-eight respondents to the survey identified 5748 priorities, which were coded into 22 themes. These covered a range of individual, social and wider socioeconomic, environmental and cultural priorities. Diet/nutrition and exercise were most commonly identified as important for health, including what needs to change to improve health. For happiness, home life and family relationships, listening to children, and education/activities were the most commonly identified. Community assets were identified as important to change for both health and happiness. From the survey response the steering group developed 27 research questions. There were mapped onto existing and planned research agendas within BiB.

**Conclusions:**

Communities identified both structural and individual factors as important priorities for health and happiness. We demonstrate how communities can be involved in priority setting using a co-productive approach in the hope this can be used as a model for others. The resulting shared research agenda will shape future research to improve the health of families living in Bradford.

**Supplementary Information:**

The online version contains supplementary material available at 10.1186/s12939-023-01881-y.

## Background

Involving the patients and the public in all aspects of the research process, often referred to as patient and public involvement engagement (PPIE) is considered best practice internationally [1]. Along with moral and ethical considerations about the importance of valuing perspectives of the final end users of research there are many other benefits. Genuine involvement of these groups can improve the quality of research, for example, by defining user-relevant questions, user friendly materials, acceptable recruitment strategies, and by helping with interpretation and dissemination of findings [2, 3].

Increasing commitment to PPIE in research has been reported [1], aided by frameworks such as the UK Standards for Public Involvement [4], with over 60 frameworks for supporting and evaluating PPIE in research having been developed and applied, including for research priority setting [5]. Effective research priority setting can help produce relevant research that meets critical evidence gaps and informs decision making to improve population health and outcomes, facilitate shared responsibility and accountability of the research agenda and improve the legitimacy of research [6].

However historically within health research, research priorities have often been set without the explicit inclusion of patients or the public [6, 7]. Heterogeneous approaches to research priority setting and “suboptimal” reporting have sometimes led to a lack of transparency including specifying who was involved in setting the priorities [8]. Priority setting which involves stakeholders such as communities, seldom heard groups and those experiencing poorer health outcomes, for example people from ethnic minority backgrounds [8, 9], can help to address inequality by ensuring that these groups voices are listened to and acted upon. Many different research priority setting frameworks exist, shaped by needs and context, with no universal or gold standard approach existing or even desirable [10].

Whilst historically priority setting may have excluded public participation, more recently approaches such as the James Lind Alliance (JLA) priority setting partnerships have been implemented. These actively involve “people with experience of the health area in question, carers and families of those affected and health and social care professionals working with patients and carers” [11]. Within these developments, priority setting activities with a child health focus have had a predominant focus on specific medical conditions or diseases, primarily in a health and care setting [12]. Whilst individual priorities around health determinants have been reported in these priority setting activities [12], and specific adaptations of the JLA to address specific topics of relevance, for example parenting [13], there is an absence of comparable priority setting research literature encompassing a broader perspective of health, particularly in a non-health care, wider determinants context.

Reporting of priority setting has also historically been suboptimal with regard to how priorities set with communities will be taken forward into active research programmes. In their qualitative study conducted with public, policy and research participants involved in priority setting exercises, Abma et.al., (2014) found four contextual factors which influenced the likelihood of shared research agendas progressing into action. These included i) organisations/researchers having a positive attitude and commitment to involving the public, ii) researchers who felt comfortable working ‘outside the box’ in relation to their specific areas of research expertise, iii) research programmes allowing adequate time and flexibility to enable effective engagement; iv) continued provision of resources, including staff time and monetary support to facilitate ongoing engagement in priority setting and research activities [6].

### Rationale for study

The current study was set in Bradford District, in the north of England which has a young and multi-ethnic population of over half a million people, one third of which are from minority ethnic communities [14]. Post-industrial urban centres, with high levels of deprivation and poor health exist alongside a two thirds rural geography. A quarter of children grow up in poverty [15] and over one third of electoral wards, local government administrative units, are amongst the 10% most deprived in England [16].

Based within the city of Bradford, Born in Bradford (BiB) is working to understand reasons for ill health and catalyse change, building on and with assets in the District, through co-production with communities and professional stakeholders. Established in 2007, it hosts three birth cohort studies, following the lives of (to date) over 40,000 Bradford residents, as well as an internationally recognised programme of applied health research with a focus on health inequalities in deprived and ethnic minority populations [17].

### Aims of current study

The aim of the current study was to report on a community led research priority setting exercise to inform a shared research agenda for BiB. This research priority setting exercise, involving communities, professional stakeholders (e.g. staff working in local government, health service, youth justice, community and voluntary sectors) and researchers, living or working in the Bradford District was undertaken to:

1. identify what is most important to keeping children and young people healthy and happy
2. co-produce a set of research priorities for happy and healthy children and young people

The aim of this paper is to describe the findings of our priority setting exercise and identify key lessons for others wishing to learn from our approach.

## Methods

To aid comprehensive reporting we have followed the REPRISE [8] guidelines for priority setting research, structuring the Methods section in-line with these guidelines.

### Context and scope

The priority setting exercise took place in Bradford District facilitated by BiB which is described in more detail in the Background section above.

BiB has a long and strong history of community engagement and co-production both with BiB families, the community and professional stakeholders [18, 19]. Examples of BiB research impact on clinical, educational and social policy include the introduction within the city of universal screening for gestational diabetes, the establishment of a regional congenital anomalies register, early life interventions for obesity prevention and physical activity, the redesign of mental health services to improve detection and support for children with autism, changes to school admission policy for children born prematurely. It has been the catalysts of over £100million investment into the city for preventive interventions to improve outcomes for pregnant women and children aged 0–3 [20], increase physical activity [21] and to reduce pollution via the design and evaluation of an ambitious clean air zone [22].

Research at BiB has grown organically responding to both research findings and emerging local issues. In autumn 2019 Bradford Institute for Health Research (BIHR), where BiB is based, became the home for two new programmes relevant to child health research, the NIHR Applied Research Collaboration (ARC) Yorkshire and Humber and ActEarly. The ARC “supports people-powered research that aims to tackle inequalities and improve health and well-being for our communities” [23]. Through ActEarly, city collaboratories have been established in Bradford District and the London Borough of Tower Hamlets “to provide research-ready, people-powered and data-linked test beds to co-produce, implement and evaluate multiple novel early life interventions to prevent disease and reduce inequalities” [24]. Collectively, with BiB, these programmes address research questions across a spectrum of health determinants, such as those described in the Dahlgren-Whitehead rainbow model [25].

This convergence of three diverse but complementary “people powered research” programmes with emphasis on co-production, where co-production in this context is defined by characteristics of practice including equality, reciprocity with individuals as agents of change [26], created an opportunity for community and professional stakeholders to shape and inform future research agendas. A research priority setting activity was proposed to identify the collective priorities for happy and healthy children. In order to truly hear from communities, we put no limits or constraints on the type of priorities or research questions that could arise from the exercise.

### Governance and team

The project was instigated by the BiB team who then convened a multidisciplinary project steering group to co-produce and oversee the project, further detail about which is provided in the stakeholders and participants section below. Once established, the steering group agreed the scope of the exercise: i) to explore what communities think is important to understand to keep children healthy and happy, and ii) and to explore what communities think needs to change improve the health and happiness of children in Bradford. A total of five meetings, hosted in community venues and lasting approximately two hours each, were held between December 2018 and January 2020.

A project team consisting of the principal investigator, research programme lead, public health specialist, study co-ordinator, research fellow and undergraduate and postgraduate students met regularly to implement the priority setting project plan. The project team included researchers with a range of skills including co-production, qualitative, quantitative, community engagement and research agenda setting expertise as well as practical public health expertise.

### Framework for priority setting

We based our priority setting process on the JLA approach [11] which was deemed suitable as it explicitly aims to bring people together on an equal footing. However this framework has been traditionally used to set research priorities in the context of clinical research and healthcare settings, working on defined topics with limited application to set general priorities in community settings. The framework involves following five stages: 1 – creating a steering group comprised of equal representation of patients, carers and clinicians to develop the project protocol, 2 – gathering evidence uncertainties in relation to the topic of interest, 3 – summarising responses to establish a long list and evidence checking to explore whether the question has already been answered, 4 – interim priority setting to establish a ‘long list’ which can be voted on in an online ranking survey, 5 – final workshop to achieve consensus on the top 10 priorities [11].

**Fig. 1:**
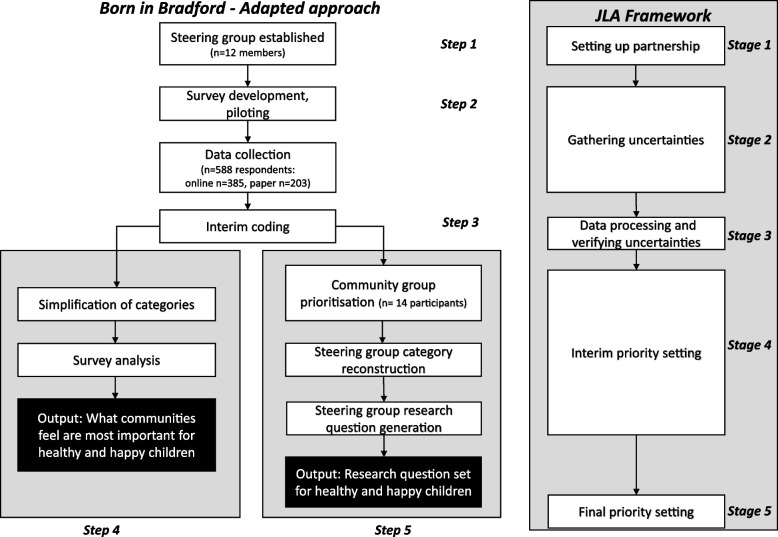
Iterative co-produced priority setting process

We amended the approach (Fig. 1). The key differences were: in JLA stage 1, in line with our ActEarly city collaboratory approach [24] our steering group was designed to include equal representation from the general public, policy/practitioners and researchers to ensure all perspectives were heard. We removed part of JLA stage 3, verifying uncertainties through evidence checking, due to the broad scope of the priority setting exercise and the fact we were interested in understanding what was important to communities. It was not practical or desirable to exclude topics that had been raised based on what was reported in existing literature.

We added a new step (step 4) and used the data to give an overview of the most frequently identified priorities identified by communities in Bradford. Through a series of steering group meetings and workshops, used the data gathered to agree a comprehensive set of research questions that reflected information gathered from communities (step 5, aligned with JLA stage 4). These final steps were the key outputs of the process. Although we had originally planned to include JLA stage 5, ranking the research questions through a consensus workshop to agree a ‘top ten’, we were unable to do so as a result of the emerging COVID-19 pandemic which coincided with the planned activities. Ethical approval for the study and survey was granted by the Chair of the Humanities, Social and Health Sciences Research Ethics Panel (reference E702) at the University of Bradford on 20/11/18.

### Stakeholders and participants

Co-production was central to the project design and activities with participation and input actively sought from the public, including children and young people; professional stakeholders such as local authority, NHS, faith groups, and community and voluntary sector staff; and researchers. Our multi-disciplinary steering group contained 12 members who were purposefully identified and invited, with a representative mix of professional stakeholders, faith, parents, voluntary and community sectors representatives and lay representatives from across Bradford District, with links to seldom heard groups and reflective of Bradford multi-ethnic population. Members were contacted and invited to join the group with remuneration provided to lay representatives in line with BiB public participation policies. At the initial meeting, participants reviewed membership and invited further members to attend to ensure all appropriate organisations from the District were adequately represented.

### Identification and collection of research priorities

#### Survey development and piloting

Questions for the research priority setting were co-produced through the steering group. An initial brainstorming activity was held to crowd source potential questions that aligned with the research aim. At subsequent meetings researchers proposed a set of questions based on the initial feedback which the steering group then reviewed and revised. Members piloted draft questions with their respective contacts and networks including with different communities in Bradford, individuals from different professional backgrounds and with researchers. BiB also sought the input and advice from its established research advisory groups including the Community Research Advisory Group, Parent Governors Group and Young Ambassadors (BiB children) whose members are familiar with providing advice and guidance to researchers. The advice and feedback from the group was that happy and healthy should be considered separately as they were likely to elicit different responses, representing different aspects of childhood. Further it was felt that respondents would have ideas of what needed to change to help increase children’s health and happiness based on personal experience and it was important to allow this to be expressed and considered as part of shaping a future research agenda.

Final refinements were made with a set of four questions agreed:

1. What things should researchers try to find out to help children be healthy?
2. What things should researchers try to find out to help children be happy?
3. What needs to change to help children be healthier?
4. What needs to change to help children be happier?

The steering group helped draft promotional and guidance materials for completing the survey with respondents asked to provide up to three responses for each question. Advice was provided on how to best present the questions in an online survey format. A further set of demographic questions were also included and whilst no questions were mandatory it was made clear that demographic questions were optional.

The potential to host the online survey in languages other than English was debated by the steering group. Ultimately it was agreed that direct engagement through face to face contacts between researchers and individuals whose first language was not English was a more appropriate means of administering the survey. This is consistent with other BiB research projects given the wide range of multiple languages spoken in the city and therefore need for multiple translations, the challenge of accurately translating questions to retain their original intent and meaning and the limited research capacity to translate answers into English for analysis.

The survey was open to all age ranges but we recognised it may not be the most appropriate method for engaging with children and young people, we therefore anticipated the majority of respondents were likely to be adults. In parallel but separate from this priority setting exercise, engagements with children and young people were also taking place across BiB research projects, most notably to shape and co-design Age of Wonder [27], the next phase of the original BiB cohort, ensuring that the voice of children and young people shape our research programme and activities.

The online survey was hosted on a secure online platform used by Bradford Teaching Hospitals NHS Foundation Trust. By completing the survey it was made clear that respondents would be consenting to provide the data for subsequent research use. No incentive or remuneration was offered to participants.

#### Data collection

The online survey was launched in March 2019 and remained open until September 2019. A short URL survey link was widely distributed and promoted across Bradford District through BiB’s social media channels, with BiB families through newsletters, promotional flyers and other opportunities with the general public as they arose such as a local radio interview and local newspaper feature. The target audience was anyone living, studying and/or working in the Bradford District, including children and young people. Steering group members widely circulated the survey link through their networks including organisations such as the Local Authority, Clinical Commissioning Group and voluntary and community services with particular links to children and young people to elicit responses from all age groups and backgrounds. Researchers also visited a local higher education college to meet with tutors as a means of generating interest and awareness in the project and to promote the survey.

Paper surveys were made available at the Bradford Royal Infirmary Glucose Test Tolerance clinic (attended by pregnant women), hospital reception and at specific public events such as the Bradford Science Festival where researchers were on hand to speak about the project. A commercial organisation was contracted to engage with the public and increase response rates by visiting a local supermarket and shopping centre locations. At further public events such as local festivals, survey forms complete with self-addressed envelopes were available to the public for completion and return at their convenience.

#### Interim coding

Data that were collected were subjected to an interim coding exercise to enable steps 4 and 5 to be run in parallel. In line with the JLA approach at this stage we wished to represent all unique priorities that were submitted by respondents to the survey. A comprehensive coding frame was thus developed inductively to represent the diversity of the data by the study co-ordinator (AR) and a community researcher (ER) who both coded all survey responses. The interim coding frame at this point had over 189 unique codes which emerged from the analytic process. These were grouped into 22 individual themes, each theme representing a distinct concept.

These findings were taken to the community consultation workshops, described further in topics/question section below. The themes and sub codes were discussed and some codes were eliminated at the first consultation as it was felt that they fitted into other themes.

#### Factors communities identified as important for happy and healthy children

In contrast to the research question set development, in analysing the factors communities identified as important for happy and healthy children we sought to identify the similarities in responses. The interim coding framework was too detailed and complex for this purpose. Consequently we developed a simplified coding framework for this analysis, informed by the work of the condensing of themes by the community and steering groups (BL,AR). This simplified coding framework was tested for adequacy on a sub-set of 100 records (BL). The remainder of the responses were coded independently by three members of the research team (AR, MW, MZ) who discussed their results with each other.

Basic descriptive analysis of the data, using coding themes, was undertaken for each question. The distribution of responses across themes was established by calculating proportions using Microsoft Excel. Themes were also ranked according to frequency to identify patterns in the types of issues most frequently identified for each question.

### Prioritization of research topics/questions and outputs

A community workshop comprised of fourteen participants and lasting approximately three hours was held in December 2019 in a community venue to prioritise the themes from the interim coding frame in to broad research priority topic areas. Participants were primarily comprised of BiB public involvement group members who were able to commit time to attend a single event in addition to two steering group members. Remuneration was provided to lay representatives in line with BiB public participation policies.

**Fig. 2:**
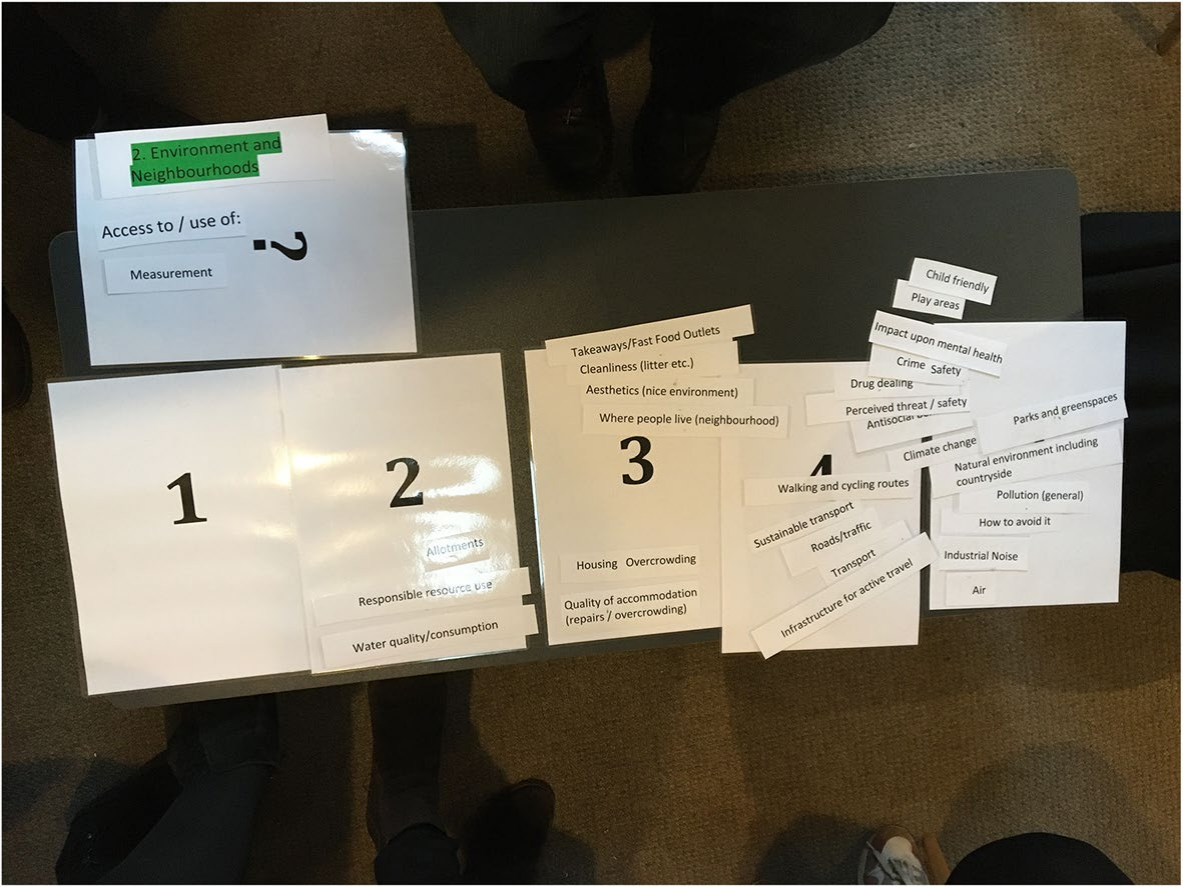
Photograph of theme prioritization for the Environment and Neighbourhoods category and sub-codes

Group discussions were facilitated to review and prioritise the themes of the coding framework. Participants were split into two groups, each reviewing a different set of themes (and their sub codes) of the framework. The themes and headings were printed and laminated and following debate placed on a priority scale of one to five (five being the highest priority, one being the lowest) in terms of the group consensus of what is of importance for healthy and happy children (Fig. 2). This prioritisation considered the themes (and sub codes) only, enabling the group to prioritise according to their discussions, without being influenced by the frequency and distribution of survey responses.

**Fig. 3:**
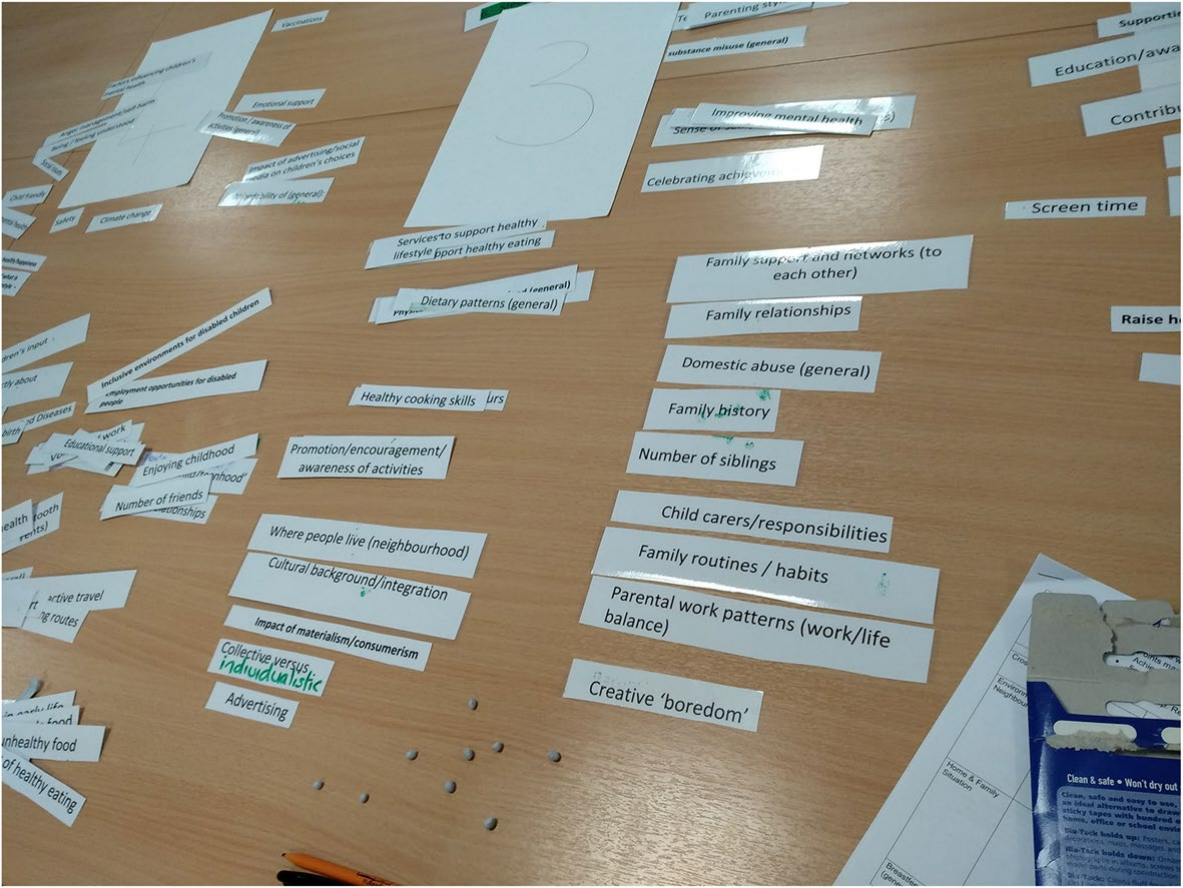
Photograph of the creation of new community generated groupings

The outputs from the community workshop were then presented at a subsequent steering group workshop in January 2020, according to the community group prioritisation. In this second workshop, participants reviewed the themes, creating new; reconstructed groups which they were felt were connected in some way, sharing similar characteristics important for happy and healthy children (Fig. 3). This led to community generated groupings established through consensus, providing a basis for research question generation.

Researchers reviewed the contents of the community generated groupings for happy and healthy children, and developed an initial set of potential research questions for discussion with the steering group. In early March 2020 the steering group reviewed and proposed amendments to the questions to make them more easily understood and accessible to the public.

### Evaluation and feedback

Dissemination of findings with stakeholders, including the public is planned in line with the BiB commitment to feedback research findings to participants, communities and policy makers.

### Implementation

Community generated research questions were mapped to the existing research agendas in BiB, ActEarly and ARC (see Additional file 1) with direct engagement with researchers planned through workshops and events to disseminate and discuss findings and in particular gaps in existing research.

### Funding and conflict of interest

Funding for staff to implement the priority setting exercise was provided via the ActEarly and ARC priority setting programmes. No conflicts of interest were declared.

## Results

### Survey findings

#### Responses and respondent demographics

Across the four questions posed, there were a total of 5748 responses (Question 1 *n* = 1583, Question 2 *n* = 1462, Question 3 *n* = 1508 and Question 4 *n* = 1195) from 588 individual respondents. Two thirds of responders (65% *n* = 385) completed the online survey and one third (35% *n* = 203) a physical survey form.

84% (*n* = 492) of responders voluntarily responded to at least one demographic question (Table 1). Of those who provided geographic information, 91% (*n* = 452) lived in the Bradford District with a broad spread of responses from Bradford’s 24 urban, rural and mixed urban/rural postcode areas. Two thirds were aged 21–50 years with four fifths being female. The representation of responders from ethnicities and cultural backgrounds other than White was higher than the wider District profile [14].

**Table 1:** Demographic profile of respondents

**Category**	**%**	**(n)**	
**Gender**		496	
Female	83	410	
Male	14	71	
Prefer to use own term	1	5	
Prefer not to say	2	10	
**Age**		492	
Under 11 years	1	5	
11–15 years	0	0	
16–20 years	10	47	
21–30 years	18	88	
31–40 years	30	147	
41–50 years	19	94	
51–60 years	14	68	
61–70 years	5	25	
71–80 years	2	12	
Over 80 years	0	1	
Prefer not to say	1	5	
**Ethnicity and cultural background**		504	**District comparison**
White	60	288	67.4%
Asian British/Asian	30	147	26.8%
Black British/Black/African/Caribbean	2	10	1.8%
Mixed/Multiple Ethnicity	4	21	2.5%
Other	3	16	1.5%
Prefer not to say		22	

A small proportion answered in the capacity of being a child or young person aged 18 or under, with parents accounting for 56% (*n* = 296) of respondents, public and professionals working with children each accounting for 28% (*n* = 145) and researchers accounting for 6% (*n* = 29).

#### What is important to keep children healthy and happy?

Table 2 presents the simplified themes and exemplar verbatim quotes. 22 different themes were identified ranging from community assets, for example green spaces and sports facilities, to environment to mental and emotional health with responses encompassing a range of individual, social and wider socioeconomic, environmental and cultural determinants. The depth and detail provided in responses varied considerably. In the diet and nutrition theme for example, responses ranged from short answers e.g. “diet”, “healthy food” or “children’s diet” through to longer responses such as “how do children who have free school meals fare in the holidays”. Similarly, in the outside theme, responses ranged from “days out” through to “more access to walk in parks, in bigger cities it appears that you have to drive everywhere to spend time outdoors”.

**Table 2:** Simplified coding themes and example responses

Theme	Theme descriptor	Exemplar responses
Access to healthcare	Access to GPS, dentist, mental health services etc	• “Easier access to help from services if needed. Children's services, health visitors” • “Effective relationship between families and primary health staff: e.g. information about inoculations” • “Whether or not they have access to health care that they can afford when they need to access it”
Community assets	More community assets/services including greens spaces, community centres, sports facilities	• “What their local area is like…facilities/green space” • “What opportunities are there for the child to play, explore the world (own environment)” • “More free out of school activities, youth clubs, theatre schools, sports for children to have somewhere safe to be out school time”
Diet and Nutrition	Anything around diet and nutrition, health eating, adverts, education around diet and nutrition. Includes breastfeeding	• “Cut sugars out of shops, why are sweet so cheap and salads and fruits expensive” • “What do families eat and what drives them to eat it?” • “Food—They can’t always have what they want and the amount they want. They need good nutrition but that doesn’t mean boring either”
Economic circumstances	Related to home life, but more specific about financial circumstances of families, poverty, housing, parents’ employment etc	• “Whether they have their basic needs fulfilled” • “Pocket money” • “More things to do outside the home, what is actually available for children to do that does not cost money”
Education and activities	School life, curriculum, extra-curricular activities	• “Why the education system fails to teach understanding and values recall” • “Reading is good for the brain, kids should do this” • “Less pressure from teachers and parent about exams. Stressed out because people around them are saying if they don’t do well in exams they will always be in poverty”
Environment	Pollution, littering, climate change, or non-specific ‘environment’	• “Road safety- to many children getting into accidents for reckless driving on main roads and local roads” • “The environment and the way we treat it” • “Safe, clean, environment( situational and on line)”
Exercise	Think children should be moving more, exercising, more physically active	• “Family situation—has the child access to healthy lifestyle from birth?” • “Would more cycle paths away from the road help children in Bradford be more active outside school/on the way to and from school” • “Bringing out the fun from asking more exercise and eating better. Role modelling from people within the support networks of the children and beyond”
Family history	More awareness of families’ specific health histories, genetic testing etc	• “Culture” • “Family life and backgrounds” • “What implications those impacts have later on in childhood/adulthood”
Health education	More health education for families, children, general population	• “Healthy definitions for different communities” • “What are the barrier to vaccination in those children who have missed their vaccines” • “Media influence on healthy living”
Home life and family relationships	A broad code encompassing a lot of aspects of home life, nurturing environments, time spent with children, family relationships	• “Family dynamics / engagement with their children” • “A supportive family, encouraging and supporting them in choices that they make and through problems that arise” • “If my parents are happy I will be happy. (financial strains/DV [domestic violence]/relationship strains) employment issues”
Listen to children	Should listen to what their priorities for health/happiness are and also listen to them to more to contribute to their happiness	• “What the child likes” • “Speak to them a lot about their issues they have” • “Adults need to stop asking other adults what makes children happy and ask the children what they want to change “
Mental and emotional health	Mental and emotional health of children and parents being monitored, explored, education and support around it	• “The mental health of children. Effects of social conditions on this.” • “Laughter” • “How to support children/encourage to feel empowered self-importance and confidence”
Outside	Just being outside more, fresh air (not related to ‘more green spaces’ etc.)	• “How much time they should have outside” • “Fresh air. Do they get to access the countryside? Are there any green spaces in Bradford they can access?” • “Attitudes to playing outside. There needs to be more notifications of where is a safe place to play or go with your family”
Parent support/input	Want more support for parents for specific education and help	• “More support for parents with poor emotional well being” • “Less stress on families—less focus on wealth- parents working too much—need family time” • “Educate parents on how to deal with challenging behaviour/free parent classes to help how we speak and treat children”
Physical health other	Anything not diet, exercise or routines related, could be non-specific too	• “What are the main obstacles to physical activity, outdoors (these are fairly well established)—more importantly—what can be done about it?” • “How children themselves cope with short and long term diseases.” • “Disabilities—background knowledge, what can be done to support these children or adults. What networks are available”
Play and Hobbies	Children should have the opportunity to play more, encourage their hobbies, creativity, things that stimulate them	• “Impact the lack of interaction opportunities with Other outside of school” • “Reasons children don't play out as much” • “More focus on experiences than things”
Routines	Sleep, hygiene, teeth brushing	• “How much sleep are they getting each night. Are the children in a routine? Do they have a set bed time?” • “Clean body and environment” • “Does a strict routine equate to a happier child or cause more stress.”
Safety	Safety of children, normally outside of the home, e.g. from cars and crime	• “Clean and safe environments (so that kids can feel comfortable playing outside)” • “The opportunity/safe environment to problem solve for themselves without contacting parents to do it for them” • “Worried parents (safety), people over worrying in stopping children being active”
Social world	Friends, social networks	• “Are they part of a group?” • “Less available opportunity to build companionship so most people feel lonely and unable to socialise and attend community events” • “Build social capital for children (sense of useful belonging and joining in)”
Technology	Concerned about social media, phone, gaming, computer use	• “The pros and cons of computing gaming and results in how this has an impact on activity levels” • “How social media has an effect on our children views and thoughts. If there should be a suggested age restriction” • “Wifi—network needs to be completely finished”
Wider society/world	Larger issues in wider society, discrimination, capitalism, consumerism, racism, cultural issues	• “More integrated and resilient communities” • “Offset poverty, generate opportunity but not just in a material or financial sense” • “Living in the world today might be more challenging but can be bright for those that apply themselves and seize the moment. We need to empower and activate kids by exposing them (whatever their backgrounds) to what it takes to live a ‘fulfilled’ life whatever that means from their perspective and that they actively aspire to this”
Other	Only use if very specific to that individual	• “Monitor access to new/available activities” • “Pre-natal experience—i.e. what was happening with their mum during gestation, particularly alcohol consumption leading to FASD [Fetal alcohol spectrum disorders]. As a foster carer this seems to affect a lot of looked-after children, but I suspect it's a broader issue, that isn't understood or picked up in children from more stable homes.” • “Better understanding in nursery settings, playgroups and in the community about ACES [Adverse childhood experiences] to inform their practice when supporting children and families”

Table 3 presents a summary of all responses by theme for each question including proportions and rank. Two distinct findings were evident in the responses received to the four questions posed. Firstly, respondents identified different factors as important for healthy or healthier children compared to those identified for happy or happier children. Secondly, the factors that respondents identified that researchers should focus on differed from those identified as needing to change. We discuss these findings in more detail below.

**Table 3:** Results and rankings by question

	**Q1** – **What things should researchers try and find out to help children be *healthy*?**			**Q2** – **What things should researchers try and find out to help children be *happy*?**			**Q3 – What needs to change to help children be *healthier*?**			**Q4 – What needs to change to help children be *happier***?		
**Category**	**%**	***n* = **	**Rank**	**%**	***n* = **	**Rank**	**%**	***n* = **	**Rank**	**%**	***n* = **	**Rank**
Access to healthcare	2%	33	16	0%	4	21	1%	21	18	1%	15	18
Community assets	3%	44	11	5%	66	7	***8%***	***126***	***3***	***12%***	***144***	***1***
Diet and Nutrition	***26%***	***410***	***1***	2%	32	13	***25%***	***378***	***1***	3%	32	14
Economic circumstances	3%	41	13	2%	29	17	3%	42	14	4%	44	13
Education and activities	5%	72	7	***10%***	***140***	***3***	***6%***	***86***	***4***	***10%***	***115***	***3***
Environment	3%	48	10	1%	18	18	4%	62	9	1%	17	17
Exercise	***12%***	***197***	***2***	2%	30	16	***10%***	***146***	***2***	2%	19	16
Family history	1%	18	22	1%	10	19	0%	2	22	0%	2	21
Health education	***5%***	***86***	***4***	1%	9	20	5%	82	6	1%	14	19
Homelife and family relationships	***8%***	***125***	***3***	***17%***	***251***	***1***	4%	60	11	***12%***	***141***	***2***
Listen to children	2%	31	17	***13%***	***196***	***2***	0%	6	20	5%	60	9
Mental and emotional Health	***5%***	***75***	***5***	***9%***	***138***	***4***	4%	65	8	***8%***	***101***	***4***
Outside	3%	49	9	3%	50	9	5%	74	7	4%	47	12
Parent support/input	4%	57	8	4%	53	8	***6%***	***85***	***5***	6%	68	7
Physical health other	1%	23	21	0%	3	22	0%	4	21	0%	1	22
Play and Hobbies	3%	42	12	8%	116	6	3%	43	13	6%	73	6
Routines	5%	75	6	2%	34	12	2%	30	16	1%	13	20
Safety	2%	24	20	2%	31	15	2%	35	15	5%	57	10
Social world	2%	36	14	***9%***	***137***	***5***	2%	29	17	***7%***	***80***	***5***
Technology	2%	35	15	3%	48	10	3%	51	12	5%	55	11
Wider society/world	2%	31	18	2%	32	14	4%	61	10	5%	65	8
Other	2%	31	19	2%	35	11	1%	20	19	3%	32	15
**Total**		**1583**			**1462**			**1508**			**1195**	

#### Healthy and healthier children

When asked about what was important for researchers to understand to keep young people healthy, a quarter of responses related to diet and nutrition (26%); exercise was the next most common response (12%), followed by home-life and family relationships (8%), health education (5%) and mental and emotional health (5%).

In relation to what should be changed to make young people healthier, diet and nutrition and exercise were still the top two responses (25% and 10% respectively), however the next most common response was in relation to having community assets (8%) followed by education and activities (6%) and parent support/input (6%).

Diet and nutrition, and exercise were identified as the two most important factors both as a research focus and of things that need to change. However the relative importance of themes differed across the two questions. For example, home-life and family relationships were ranked as the 3^rd^ most frequent theme with regards to understanding how to keep children healthy, however, was ranked the 12^th^ most frequent with regards to what needs to change. Community assets were the 3^rd^ most frequent theme identified in regard to what needs to change to keep children healthy but were the 11^th^ most frequently identified theme in relation to what researchers need to understand to keep children healthy.

#### Happy and happier children

When asked about what was important for researchers to understand to keep young people happy, home life and family relationships (17%) was the most common response, followed by listen[ing] to children (13%), education and activities (10%), mental and emotional health, and social world (for example friends and social networks) (both 9%). In relation to what should be changed to make young people happier, community assets and home life and family had the same proportion of responses (both 12%), followed by education and activities (10%), mental and emotional health (8%) and social world (7%).

Factors identified in both questions remained broadly similar and shared similar ranking with a shared emphasis on relationships (both family and social relations), the importance of education and activities and mental and emotional health. Community assets, of a lower rank for researcher focus, were identified as the top factor that needed to change for happier children. Interestingly listen[ing] to children was deemed less important.

#### Comparison between priorities for healthy children vs. happy children

**Fig. 4:**
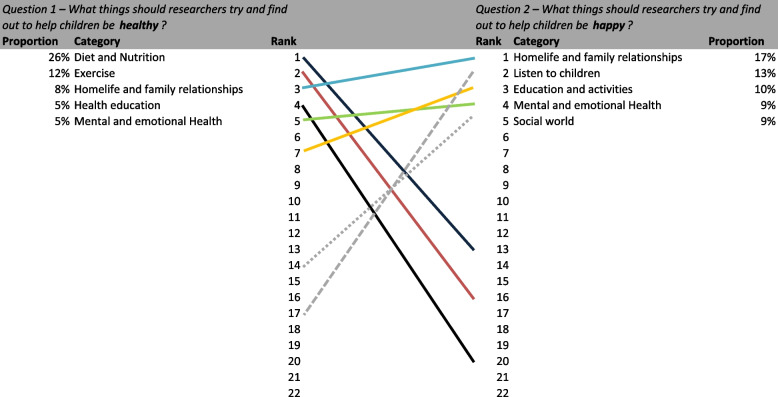
Ranking comparison of healthy (Q1) vs. happy (Q2)

**Fig. 5:**
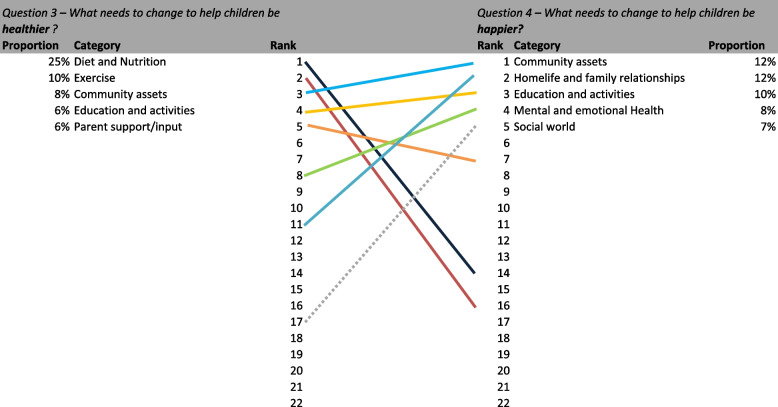
Ranking comparison of healthier (Q3) vs. happier (Q4)

There were clear differences in the types of themes that were rated as most important for keeping children healthy vs. keeping children happy (Figs. 4 and 5). The most favoured factors for keeping children healthy had a greater association with physical health and circumstances e.g. diet and nutrition, exercise etc. In contrast, responses relating to children being happy had a greater focus on relationships (both with families and peers), mental and emotional health, education and activities, and social connectedness. Home life and family relationships, education and activities, mental and emotional health and parental support and input were identified as important for both but generally had higher rankings for keeping children happy compared with keeping children healthy.

### Research question generation

The three stage research question development process (Fig. 1 – Step 5) led to the generation of research questions (Table 4) informed by the collective experience, expertise, motivations, beliefs and insights of group members. The community group and steering group workshops prioritised and reordered the interim coding framework into new groups which identified relationships between factors important for happy and healthy children. 189 codes from 22 themes were reviewed and placed into 29 new groups.

**Table 4:** Research questions for happy and healthy children

Steering group questions (Note that these are in no particular order)
1. How can we ensure health services are appropriate for community needs and accessed by those that need them?
2. What types of services (voluntary/cultural/youth) are needed to promote health and wellbeing?
3. How can we optimise a healthy diet?
4. What are the barriers to a healthy lifestyle (individual, community, structural)?
5. How does the quality of people’s housing affect their health?
6. How does children’s educational experience impact on their health and wellbeing?
7. How can we reduce exposure to pollution?
8. How best can we improve sustainable travel and encourage active travel?
9. How do we encourage children to be physically active?
10. What are the barriers that stop children from being physical active?
11. What elements of a child’s home environment are most important for health and wellbeing?
12. How do family relationships impact on children’s health and wellbeing?
13. How can we ensure their voices are heard and can influence their future?
14. What are the key issues facing children in terms of their mental health and what can we do about them?
15. How does perception of pressure to succeed impact on health and wellbeing?
16. What is childhood and how does it affect health and wellbeing?
17. How can we ensure access/encourage to high quality natural environments?
18. What is needed to understand how to support or improve parenting skills?
19. What is the impact of vaccinations on children’s health (upto date, barriers, positive messages)?
20. What is important for health and health conditions?
21. What are the barriers (individual, community, organisational environments) that stop people leading healthy lifestyles?
22. What is important for a healthy mouth for children?
23. How do children’s peer and social relationships affect their health and wellbeing?
24. What is the impact of screen time on children’s health?
25. How we can encourage different ages and communities to work together?
26. How does consumerism effect our health and wellbeing?
27. How do we build inclusive environments for children regardless of culture, ethnicity, disability and background?

The use and reconstruction of the interim coding framework in this way meant that equal consideration was given to a response code independent of the frequency or distribution of individual responses. Consequently equal weighting and consideration was applied to responses to all four questions, providing a holistic view of health and happiness. Potentially important themes which had limited number of responses were considered equally with those themes with a high proportion of responses, in line with other priority setting exercise frameworks [11].

Researchers proposed an initial question set based on the 29 community led groupings that emerged from the community group prioritisation workshop and steering group theme reconstruction workshop. The steering group edited and further developed these questions, simplifying language into a form that would likely be understood by a wider, non-academic audience into a final set of 27 questions.

## Discussion

In this community led priority setting study, we identified key areas that communities think are important for researchers to focus on to keep children healthy and happy, and what needs to change to improve levels of health and happiness in the District. From this work, a community steering group generated 27 research questions for researchers to consider in the future and understanding of what people think is important for healthy and happy children and young people in Bradford District. Whilst others have similarly applied and adapted priority setting approaches for child health [12, 13] our study is novel in that its focus was to identify whole community priorities from a multi-ethnic population with no limits on topics that could be identified and to use these to develop a shared research agenda for the District in relation to children’s health.

### Happy and healthy children

In general, different factors were rated as important for keeping children healthy compared with keeping children happy. By far the most prevalent issues in relation to keeping children healthy centred on diet and nutrition (including concepts such as healthy eating, advertising, and education) and exercise. These were also rated most frequently in relation to what needs to change to make children healthier. In contrast, in relation to what keeps children happy issues such as home life and family relationships (having a nurturing environment and positive relationship), listen[ing] to children (finding out about their priorities), and education and extra-curricular activities were identified as important. When thinking about what needs to change to make children healthier and happier, structural factors such as community assets (having a good level of assets or services including green spaces, community centres or sports facilities) and education/extracurricular activities were raised as important.

Responses encompassed the holistic view of health defined by the World Health Organization as “a state of complete physical, mental and social well-being and not merely the absence of disease or infirmity” [28] adding additional dimensions to other priority setting exercises which have had a condition/disease focus [12]. A range of determinants (i.e. individual, social and wider socioeconomic, environmental and cultural) were evident across different response themes highlighting the breadth of thinking amongst respondents about what are important factors for healthy and happy children, consistent with other studies of people’s perceptions of health determinants [29].

Diet and nutrition and exercise were key factors raised in relation to keeping children healthy. In Bradford district, 22.5% of children are obese or overweight at age 4–5, and this figure rises to 37.9% by age 10–11, with rates at ages 10–11 continuing to rise [30]. Sedentary activity is also a significant issue adversely affected by the COVID-19 pandemic when only one quarter of children were found to be sufficiently physically active [31].

For happiness, communities placed priorities on home-life and family relationships, of listening to children and the importance of the education sector. A recent study conducted in Bradford of 15,641 children aged 7–10 found that 31% self-reported one or more vulnerability in relation to subjective wellbeing (for example, keeping worries to yourself, feeling sad, never happy, cannot work out what do with things are hard, always ill or unwell) [32]. In the same study 13% reported that they didn’t like school and 5% reported that they don’t get on well with their family. The findings from the current study suggest that these vulnerabilities are issues of shared concern with the wider population.

It was interesting to note the emphasis placed on structural factors such as community assets and education in relation to making improvements. There is increasing emphasis on this with the academic and practice partnership of ActEarly specifically focussing on identifying, co-producing and implementing and evaluating system-wide interventions [24].

### Formation of research questions

The 27 research questions of importance to communities living in Bradford identified by the community steering group encompassed a range of determinants of health from individual to system level influences. The formation of these questions in communities owns voices allows them a form of agency over the nature and direction of activities carried out by researchers in the city. The questions provide a start point for researcher, public and stakeholder conversations which are built around community conception of factors influencing health e.g. “what is important for a health mouth for children” rather than a researcher focussed perspective of “how can we reduce dental caries”.

The formation of these questions was an interactive and iterative process, sparked by discussion on emerging findings and involved connecting issues and factors in a way meaningful from a public perspective, recognising inter-dependence and cross linkages absent in a purely descriptive theme based framework. This would have been impossible for a research team to do in isolation. However, due to the process, some of the ways in which the questions are worded are broad in contrast to other JLA priority setting partnerships, for example for research from conception to 2 years [13] and preventative care research [33], and much less specific than focussed priority setting for example paediatric inflammatory bowel disease [34]. We therefore recommend that they are used as a starting point for further exploration of the key factors important for these different themes of inquiry. Using the healthy mouth example, researchers should work with communities to determine a focus on individual behaviours (for example skills in parental supervised brushing) or training of health professionals.

### Co-production

We followed a truly co-productive approach to the identification of research priorities and the formation of key research questions, with greater involvement than the majority of other priority setting studies [35]. While some have observed a “dark side” to co-production [36] the challenges noted were not experienced in this project. The steering group provided valuable direction and advice to researchers with high levels of participation from individuals representing different organisations and communities. Meetings were held in community venues with members guiding and constructively challenging researchers through the project. It was noted however, that despite offering to cover childcare costs, parents with young children at home still struggled to participate due to a lack of childcare opportunities.

Whilst the steering group members knew each other and had worked together previously, the community group participants had less established relationships with each other. Two facilitators coordinated the discussions of the community group though it was observed that some group members dominated the discussions in one group, whilst participation was more equal in the second group. When working in this manner it is important to be responsive to group dynamics and skilful facilitation is required to ensure that certain voices do not dominate and to avoid ‘presence without voice, and voice without influence’ [37].

### Strengths and limitations

We used a co-productive approach to identify research priorities from multi-ethnic communities living in the Bradford District adopting a holistic approach to health and wellbeing. Our community steering group was the driving force behind the entire process. This partnership was only possible due to the time BiB has spent creating long-term and trusted partnerships with community organisations across the city. Our priority setting survey reached a broad, multi-ethnic sample of individuals across the Bradford District. By reporting our approach and findings against the REPRISE guidelines [8] we hope to aid replicability in other settings.

The process itself shifted power to the community with no distinction made between public, professionals or researcher responses. The community and steering group workshops continued this shift with the researcher’s role being that of facilitator. The input and revision of researcher generated questions by the steering group created simpler and more accessible research questions that could be shared with the community. Steering group dynamics, through established relationships, enabled equal participation and therefore different perspective inputs in this question generation.

It is also worth noting that representation from minority ethnic communities was higher than the wider district profile suggesting that individuals from different communities were able to participate despite the survey only being available in English. The notable exception is likely insufficient representation of individuals from Eastern European backgrounds where representation from White non-English/Welsh/Scottish/Northern Irish backgrounds was limited. We used a combination of remote and in-person approaches to collecting data including the online survey and a presence at community events to try and ensure we did not exclude key population groups.

Our study did have some limitations. There was limited detail that we could capture on community preferences in our brief survey. Some responses were brief which made it hard to understand nuances in individuals’ priorities. However, the aim of the exercise was to identify broad priorities and we envisage that further topic specific work would be necessary with communities to further refine topics of interest. Whilst the profile of our respondents was not fully representative of the Bradford District our diverse steering group provided some mitigation to this and we have a greater understanding of populations and communities where further engagement is needed.

We sought to reduce researcher influence for specific steps of the process, orientating their role as one of undertaking activities on behalf of and under the direction of the steering group but it is unlikely this influence was completely eliminated. For example, the development of the original survey questions by researchers was based on initial direction from the steering group and was subsequently reviewed and revised by the steering group but this oversight could still have been influenced and guided by the input and role of the researcher in formulating the initial questions.

To be inclusive and ensure as wider response and representation as possible we employed different approaches to data collection, namely paper and online surveys, which whilst increasing the number of responses, may have themselves influenced the content for example where assistance was provided for non-English speakers. Representation of children and young people themselves was extremely limited with parallel interactions using more appropriate methods taking place within other BiB projects. This does create a challenge for researchers and community groups with regard to aligning and triangulating insight and priorities potentially generated from different populations using different methodologies.

This research priority setting activity should not be viewed in isolation as a singular or definitive event but one of a number of ways in which research agendas are built and developed as an ongoing process. Research happens in a dynamic world and the dramatic changes in circumstances over the course of the COVID-19 pandemic, for example the reduction in children being sufficiently physically active (69%) pre-COVID-19 compared with the first lockdown (29%) [31], means that priorities should be continually reappraised. The list of research priorities are a reflection of a pre-COVID-19 world, and it is likely a range of additional priorities focusing specifically on the pandemic would be apparent if the process was repeated today. Our own research conducted post pandemic as part of the Bradford COVID-19 Scientific Advisory group [38] with communities has found issues of vaccine hesitancy [39, 40], adolescent mental health, food and financial insecurity [41]. Nevertheless, our research priorities are still of value. Many of the health issues and inequalities experienced by communities have been exacerbated by the pandemic and will still need addressing after the pandemic subsides.

### Implications for research, policy and practice

Findings and the mapping of research questions to the existing ARC and ActEarly research portfolios will be actively disseminated and discussed within these research groups. The broad nature of the questions means that they will provide the strategic framework and steer for future research within our projects. Through continuous dialogue with communities, with the application of different methods and approaches relevant to different populations, we will be able to triangulate findings to identify shifts or new, emergent research priorities, ensuring that our research remains relevant and responsive to changing needs and interests.

The research questions holistic nature extends their relevance beyond traditional research establishments, spheres and scientific disciplines. Many of the identified research questions are already being actively addressed not only by partnerships with policy makers in ARC and ActEarly but also independently by key stakeholder such as the Local Authority. The research questions present opportunities to further engage and discuss findings and next steps with policy makers and communities.

Whilst the primary function of the activity was to guide future research activities the findings also have important utility for both policy and practice. For policy, the findings provide a holistic view of areas where perceived changes are needed to enable children and young people to be healthier and happier, providing useful evidence for local District and service plans and policies. For practice, the findings also provide insights and evidence of support for potential interventions being planned within the city such as school streets (closure of school roads to traffic during commuting times), green infrastructure improvements or clean air zones. Findings will be actively shared with professional stakeholders and research groups to inform future practice. They will also be shared with the public through BiB’s extensive engagement and communications infrastructure.

## Conclusion

This is one of the first studies which has applied a community led co-produced research priority setting approach, that has engaged a significant number of participants from across a whole District, to develop a holistic set of community generated research questions focussed on the health and wellbeing of children and young people. Additionally, our findings also help us to understand not only what communities think is important for happy and healthy children, but also what needs to change. Our novel methodology, reported against the REPRISE guideline, equalised the power between the public, researcher and professional stakeholders and developed a question set that crosses traditional research institution boundaries in terms of scope. Mapping these findings to our existing ARC and ActEarly research portfolios has illustrated that much of our research aligns with community priorities but also presents new insights and challenges for our future research agenda. These community priorities are not static and with the onset of the COVID-19 pandemic, the need for research priority setting to be viewed as an ongoing activity is never more apparent.

## Supplementary Information

**Additional file 1.**

## Data Availability

Anonymised data is available on request via the corresponding author.

## Abbreviations

ARC: Applied Research Collaboration Yorkshire and Humber
BiB: Born in Bradford
BIHR: Bradford Institute for Health Research
JLA: James Lind Alliance
PPIE: Patient and public involvement engagement
REPRISE: Reporting guideline for priority setting of health research

## References

[1] Staniszewska S, Denegri S, Matthews R, Minogue V (2018). Reviewing progress in public involvement in NIHR research: developing and implementing a new vision for the future. BMJ Open..

[2] Brett J, Staniszewska S, Mockford C, Herron-Marx S, Hughes J, Tysall C (2014). Mapping the impact of patient and public involvement on health and social care research: a systematic review. Heal Expect.

[3] Blackburn S, McLachlan S, Jowett S, Kinghorn P, Gill P, Higginbottom A (2018). The extent, quality and impact of patient and public involvement in primary care research: a mixed methods study. Res Involv Engagem.

[4] NIHR. UK Standards for Public Involvement. 2019 [cited 19 Jan 2022]. Available from: https://drive.google.com/file/d/1U-IJNJCfFepaAOruEhzz1TdLvAcHTt2Q/view.

[5] Greenhalgh T, Hinton L, Finlay T, Macfarlane A, Fahy N, Clyde B (2019). Frameworks for supporting patient and public involvement in research: systematic review and co-design pilot. Heal Expect.

[6] Abma TA, Pittens CACM, Visse M, Elberse JE, Broerse JEW (2015). Patient involvement in research programming and implementation: A responsive evaluation of the Dialogue Model for research agenda setting Patient involvement in research programming and implementation: A responsive evaluation of the Dialogue Model for research agenda setting T A Abma et al. Heal Expect.

[7] Pratt B (2021). Sharing power in global health research: an ethical toolkit for designing priority-setting processes that meaningfully include communities. Int J Equity Health.

[8] Tong A, Synnot A, Crowe S, Hill S, Matus A, Scholes-Robertson N (2019). Reporting guideline for priority setting of health research (REPRISE). BMC Med Res Methodol.

[9] Iqbal H, West J, Haith-Cooper M, McEachan RRC (2021). A systematic review to identify research priority setting in Black and minority ethnic health and evaluate their processes. PLoS One.

[10] Viergever RF, Olifson S, Ghaffar A, Terry RF (2010). A checklist for health research priority setting: nine common themes of good practice. Heal Res Policy Syst.

[11] James Lind Alliance. The James Lind Alliance Guidebook. 2021 [cited 19 Jan 2022]. Available from: www.jla.nihr.ac.uk.

[12] Modanloo S, Correll Q, Correll R, Major N, Quinlan M, Reszel J, et al. Identifying research priorities with children, youth, and families: A scoping review. J Child Heal Care. 2023. 10.1177/13674935231151748. [cited 1 Mar 2023]. 10.1177/13674935231151748 PMC11459867 36647285

[13] Brockway ML, Keys E, Bright KS, Ginn C, Conlon L, Doane S (2021). Top 10 (plus 1) research priorities for expectant families and those with children to age 24 months in Alberta, Canada: results from the Family Research Agenda Initiative Setting (FRAISE) priority setting partnership project. BMJ Open..

[14] City of Bradford Metropolitan District Council. Demographics of Bradford District The Population of Bradford District Chapter 1 Date. Joint Strategic Needs Assessment. 2020.

[15] Office for Health Improvement and Disparities. Public Health Outcomes Framework. 2022 [cited 2 Feb 2022]. Available from: https://fingertips.phe.org.uk/profile/public-health-outcomes-framework/data#page/3/gid/1000041/pat/6/par/E12000003/ati/402/are/E08000032/iid/93701/age/169/sex/4/cat/-1/ctp/-1/yrr/1/cid/4/tbm/1.

[16] City of Bradford Metropolitan District Council. Indices of deprivation 2019. 2019 [cited 2 Feb 2022]. p. 2. Available from: https://ubd.bradford.gov.uk/media/1533/indices-of-deprivation-2019-on-the-day-alert.pdf.

[17] Born In Bradford. [cited 1 Mar 2023]. Available from: https://borninbradford.nhs.uk/.

[18] Bird PK, McEachan RRC, Mon-Williams M, Small N, West J, Whincup P (2019). Growing up in Bradford: Protocol for the age 7–11 follow up of the Born in Bradford birth cohort. BMC Public Health..

[19] McEachan RRC, Dickerson J, Bridges S, Bryant M, Cartwright C, Islam S (2020). The Born in Bradford COVID-19 Research Study: Protocol for an adaptive mixed methods research study to gather actionable intelligence on the impact of COVID-19 on health inequalities amongst families living in Bradford. Wellcome Open Res..

[20] Dickerson J, Bird PK, McEachan RRC, Pickett KE, Waiblinger D, Uphoff E (2016). Born in Bradford’s Better Start: An experimental birth cohort study to evaluate the impact of early life interventions. BMC Public Health..

[21] Hall J, Bingham DD, Seims A, Dogra SA, Burkhardt J, Nobles J (2021). A whole system approach to increasing children’s physical activity in a multi-ethnic UK city: a process evaluation protocol. BMC Public Health..

[22] Wright J, McEachan R, Mathai M. Why is the Born in Bradford cohort study important for child health? Arch Dis Child. 2021 [cited 19 Jan 2022]; Available from: https://adc.bmj.com/content/early/2021/03/11/archdischild-2020-321231. 10.1136/archdischild-2020-321231 33712432

[23] NIHR Applied Research Collaboration Yorkshire and Humber. NIHR Applied Research Collaboration Yorkshire and Humber. 2022 [cited 19 Jan 2022]. Available from: https://www.arc-yh.nihr.ac.uk/.

[24] Wright J, Hayward AC, West J, Pickett K, McEachan RM, Mon-Williams M (2019). ActEarly: a City Collaboratory approach to early promotion of good health and wellbeing. Wellcome Open Res..

[25] Dahlgren G, Whitehead M. Policies and strategies to promote social equity in health. 2007 [cited 2 Feb 2022]. Available from: https://www.iffs.se/media/1326/20080109110739filmz8uvqv2wqfshmrf6cut.pdf.

[26] Islam S, Small N. An annotated and critical glossary of the terminology of inclusion in healthcare and health research. Res Involv Engagem. 2020 [cited 19 Jan 2022];6(1). Available from: https://pubmed.ncbi.nlm.nih.gov/32337067/. 10.1186/s40900-020-00186-6 PMC7171766 32337067

[27] Dogra SA, Lightfoot K, Kerr R, Hall J, Joseph O, Siddig N, et al. STUDY PROTOCOL Born in Bradford Age of Wonder cohort: A protocol for qualitative longitudinal research [version 1; peer review: 1 approved]. 2022 [cited 1 Mar 2023]; Available from: 10.12688/wellcomeopenres.18096.1. 10.12688/wellcomeopenres.18096.3 PMC10362373 37485293

[28] Organization. WH. Constitution of the World Health Organization. 1946 [cited 19 Jan 2022]. Available from: https://www.who.int/about/governance/constitution.

[29] Zahra A, Lee EW, Sun LY, Park JH (2015). Perception of Lay People Regarding Determinants of Health and Factors Affecting It: An Aggregated Analysis from 29 Countries. Iran J Public Health..

[30] City of Bradford Metropolitan District Council. Living Well: Overweight and Obesity Chapter 4 Date. Joint Strategic Needs Assessment. 2019 [cited 25 Jan 2022]. Available from: https://jsna.bradford.gov.uk/documents/Peoplearelivingtheirliveswellandageingwell/4.2LIfestyleFactors/OverweightandObesity.pdf.

[31] Bingham DD, Daly-Smith A, Hall J, Seims A, Dogra SA, Fairclough SJ (2021). Covid-19 lockdown: Ethnic differences in children’s self-reported physical activity and the importance of leaving the home environment; a longitudinal and cross-sectional study from the Born in Bradford birth cohort study. Int J Behav Nutr Phys Act..

[32] Pickett KE, Ajebon M, Hou B, Kelly B, Bird PK, Dickerson J, et al. Vulnerabilities in child wellbeing among primary school children: a cross-sectional study in Bradford, UK. medRxiv. 2021 [cited 25 Jan 2022];2021.01.10.21249538. Available from: 10.1101/2021.01.10.21249538v1. 10.1136/bmjopen-2021-049416 PMC9247690 35772827

[33] Lavigne M, Birken CS, Maguire JL, Straus S, Laupacis A (2017). Priority setting in paediatric preventive care research. Arch Dis Child..

[34] Grant A, Crane M, Laupacis A, Griffiths A, Burnett D, Hood A (2019). Engaging Patients and Caregivers in Research for Pediatric Inflammatory Bowel Disease: Top 10 Research Priorities. J Pediatr Gastroenterol Nutr..

[35] Grill C. Involving stakeholders in research priority setting: a scoping review. Res Involv Engagem. 2021 [cited 2 Feb 2022];7(1). Available from: https://www.ncbi.nlm.nih.gov/pmc/articles/PMC8555197/. 10.1186/s40900-021-00318-6 PMC8555197 34715932

[36] Oliver K, Kothari A, Mays N (2019). The dark side of coproduction: Do the costs outweigh the benefits for health research?. Heal Res Policy Syst..

[37] Pratt B (2019). Towards inclusive priority-setting for global health research projects: recommendations for sharing power with communities. Health Policy Plan..

[38] Bradford Institute for Health Research. Bradford’s COVID Scientific Advisory Group (CSAG). 2021 [cited 25 Jan 2022]. Available from: http://bit.ly/37whJsn.

[39] Lockyer B, Islam S, Rahman A, Dickerson J, Pickett K, Sheldon T (2021). Understanding COVID-19 misinformation and vaccine hesitancy in context: Findings from a qualitative study involving citizens in Bradford. UK. Heal Expect..

[40] Dickerson J, Lockyer B, Moss RH, Endacott C, Kelly B, Bridges S (2021). COVID-19 vaccine hesitancy in an ethnically diverse community: descriptive findings from the Born in Bradford study. Wellcome Open Res..

[41] Dickerson J, Kelly B, Lockyer B, Bridges S, Cartwright C, Willan K (2021). Experiences of lockdown during the Covid-19 pandemic: descriptive findings from a survey of families in the Born in Bradford study. Wellcome Open Res.

