# Supplementary material for: People powered research: what do communities identify as important for happy and healthy children and young people? A multi-disciplinary community research priority setting exercise in the City of Bradford, United Kingdom (UK)
Source: Int J Equity Health. 2023 Apr 25;22:71. doi: 10.1186/s12939-023-01881-y (PMC10125860; doi:10.1186/s12939-023-01881-y)
Supplement: Supplementary file 1 — Additional file 1. [file 12939_2023_1881_MOESM1_ESM.doc]

**Supplementary File 1:**

Mapping of ARC and ActEarly Research Programmes to Research Question Set

| **Questions (in no particular order)** | **Alignment** | |
| --- | --- | --- |
|  | **ARC** | **ActEarly** |
| 1. How can we ensure health services are appropriate for community needs and accessed by those that need them? | Best Start, Health Equity & Minority Ethnic Health | Healthy Livelihoods and Healthy Schools |
| 2. What types of services (voluntary/cultural/youth) are needed to promote health and wellbeing? | - | - |
| 3. How can we optimise a healthy diet? | Healthy Bodies | Food and Nutrition |
| 4. What are the barriers to a healthy lifestyle (individual, community, structural)? | All topics | All themes |
| 5. How does the quality of people’s housing affect their health? | Healthy Places & Health Equity | Healthy Places |
| 6. How does children’s educational experience impact on their health and wellbeing? | Healthy Schools & Systems Change | Healthy Learning |
| 7. How can we reduce exposure to pollution? | Healthy Places | Healthy Places |
| 8. How best can we improve sustainable travel and encourage active travel? | Healthy Places | Healthy Places, Physical Activity & Play |
| 9. How do we encourage children to be physically active? | Healthy Bodies | Healthy Places, Physical Activity & Play |
| 10. What are the barriers that stop children from being physical active? | Healthy Bodies | Healthy Places, Physical Activity & Play |
| 11. What elements of a child’s home environment are most important for health and wellbeing? | Best Start | Healthy Livelihoods, Healthy Places |
| 12. How do family relationships impact on children’s health and wellbeing? | Best Start | - |
| 13. How can we ensure their voices are heard and can influence their future? | Systems Change | Co Production |
| 14. What are the key issues facing children in terms of their mental health and what can we do about them? | Healthy Schools, Systems Change | Healthy Learning |
| 15. How does perception of pressure to succeed impact on health and wellbeing? | - | - |
| 16. What is childhood and how does it affect health and wellbeing? | All topics | - |
| 17. How can we ensure access/encourage to high quality natural environments? | Healthy Places | Healthy Places |
| 18. What is needed to understand how to support or improve parenting skills? | Best Start | - |
| 19. What is the impact of vaccinations on children's health (upto date, barriers, positive messages)? | - | - |
| 20. What is important for health and health conditions? | All topics | All themes |
| 21. What are the barriers (individual, community, organisational environments) that stop people leading healthy lifestyles? | All topics | All themes |
| 22. What is important for a healthy mouth for children? | Oral Health | - |
| 23. How do children’s peer and social relationships affect their health and wellbeing? | Healthy Schools, Systems Change | Healthy Learning |
| 24. What is the impact of screen time on children’s health? | Healthy Bodies | - |
| 25. How we can encourage different ages and communities to work together? | - | - |
| 26. How does consumerism effect our health and wellbeing? | - | - |
| 27. How do we build inclusive environments for children regardless of culture, ethnicity, disability and background? | - | - |
